# Transcriptomic studies of systemic lupus erythematosus

**DOI:** 10.1186/s41232-021-00161-y

**Published:** 2021-04-09

**Authors:** Masahiro Nakano, Yukiko Iwasaki, Keishi Fujio

**Affiliations:** Department of Allergy and Rheumatology, Graduate School of Medicine, The University of Tokyo, 7-3-1, Hongo, Bunkyo-ku, Tokyo, 113-8655 Japan

**Keywords:** Systemic lupus erythematosus, Lupus nephritis, Transcriptome, Review

## Abstract

The management of systemic lupus erythematosus (SLE) remains challenging for clinicians because of the clinical heterogeneity of this disease. In attempts to identify useful biomarkers for the diagnosis of and treatment strategies for SLE, previous microarray and RNA sequencing studies have demonstrated several disease-relevant signatures in SLE. Of these, the interferon (IFN) signature is complex, involving IFNβ- and IFNγ-response genes in addition to IFNα-response genes. Some studies revealed that myeloid lineage/neutrophil and plasma cell signatures as well as the IFN signature were correlated with disease activity, lupus nephritis, and complications of pregnancy, although some of these findings remain controversial. Cell-type-specific gene expression analysis revealed the importance of an exhaustion signature in CD8^+^ T cells for SLE outcome. Recent single-cell RNA sequencing analyses of SLE blood and tissues demonstrated molecular heterogeneity and identified several distinct subpopulations as key players in SLE pathogenesis. Further studies are required to identify novel treatment targets and determine precise patient stratification in SLE. In this review, we discuss the findings and limitations of SLE transcriptomic studies.

## Background

Systemic lupus erythematosus (SLE) is a systemic autoimmune disease characterized by the breakdown of self-tolerance and production of autoantibodies [1]. The treatment goal is to achieve remission or a low disease activity state and to prevent flares while maintaining the lowest possible dose of glucocorticoids [2–4]. However, the clinical management of SLE remains challenging because of its diverse clinical manifestations and unpredictable disease course. To date, genome-wide association studies (GWASs) have identified more than 100 susceptibility loci in SLE, including genes related to clearance of immune complexes or waste, nucleic acid sensing and interferon (IFN) signaling, and lymphocyte activation pathways [5–8]. Although GWASs have proved to be a powerful tool to evaluate the associations between genetic loci and traits, the precise mechanism of how these genetic changes cause disease remains unknown. In this context, transcriptome analyses in SLE patients could help detect novel treatment targets, as well as biomarkers for SLE pathogenesis, disease activity, and patient stratification based on gene expression profiles. Here, we reviewed previous transcriptomic studies of SLE including microarray, RNA sequencing (RNA-seq), and recent single-cell RNA-seq (scRNA-seq) analyses and investigated which cell subpopulations and immunological pathways play significant roles in SLE pathogenesis.

## The IFN signature: its complexity and correlation with SLE clinical features

Aberrant activity of the innate immune system has been implicated as a key player in SLE pathogenesis [1, 5]. Early microarray studies of SLE revealed distinct blood transcriptional signatures associated with type I IFN [9–11], and subsequent studies showed a correlation between these type I IFN signatures and disease activity [12, 13]. In 2008, Pascual and colleagues performed modular analysis of whole-blood microarray data obtained from 239 individuals including 40 pediatric SLE patients [13]. Among 28 transcriptional modules containing coordinately expressed genes obtained from multiple disease data sets, 11 including an IFN-inducible module were significantly upregulated or downregulated in SLE. Moreover, the IFN-inducible module was positively correlated with SLE disease activity. However, other groups did not validate the association between type I IFN activation and SLE disease activity in longitudinal studies [14–16]. Pascual and colleagues then applied their modular analysis approach in a subsequent study of 157 whole blood samples from 62 SLE patients; they identified 3 distinct IFN-annotated modules (M1.2, M3.4, and M5.12), which showed different activation thresholds (M1.2 < M3.4 < M5.12) [17]. Longitudinal analyses revealed that M1.2 was stable, whereas M3.4 and M5.12 were relatively variable over time in single patients. They also stratified patients based on the activity of each module, and moderate/strong modular scores (2 or 3 active) were associated with higher titers of anti-double-stranded DNA (anti-dsDNA) antibodies and lower lymphocyte counts compared with absent/mild modular scores (0 or 1 active). Among the 3 modules, M3.4 and M5.12 were correlated with cutaneous flares, whereas M5.12 was correlated with renal flares. Interestingly, using other public data sets, the authors also showed that M3.4 and M5.12 can be induced by IFNβ and IFNγ. This study revealed the complexity of the IFN signature and the importance of the transcripts selected to determine the IFN signature score.

Other groups also attempted to categorize the IFN signature using various methods. Using factor analysis of 31 IFN-stimulated genes (ISGs) from 3 IFN-annotated modules, El-Sherbiny et al. developed a 2-score system: IFN score A and B [18]. In sorted cell subsets (monocytes, T cells, natural killer [NK] cells, naive B cells, memory B cells, and plasmablasts), the greatest differences in both scores between SLE and healthy controls were seen in monocytes. Compared with healthy controls, IFN score A was increased in SLE patients only, whereas IFN score B was increased in both SLE and rheumatoid arthritis (RA) patients. In the SLE patients, both scores were correlated with mucocutaneous and hematological activities, but not musculoskeletal activity. A subsequent study by Yusof et al. in 118 individuals at risk of an autoimmune disease, as defined by anti-nuclear antibody positivity, showed that both scores, especially IFN score B, predicted progression to an autoimmune disease (14 SLE and 5 primary Sjogren’s syndrome cases) [19].

Catalina et al. defined IFNα2-, IFNβ1-, IFNω1-, and IFNγ-induced signatures in in vitro-stimulated normal human peripheral blood mononuclear cells (PBMCs) [20]. By applying gene set variation analysis to multiple publicly available datasets, they showed that all four IFN signatures, especially the IFNβ1 signature, were highly enriched in skin and synovium of SLE patients, whereas all signatures were relatively less enriched in the kidney (both the glomerulus and tubulointerstitium) of lupus nephritis (LN) patients. They showed that the IFN signature was especially overexpressed in monocytes compared with CD4^+^T and B cells in SLE patients. It was concluded that persistent overexpression of IFN signatures in monocytes in inactive SLE, defined by an SLE disease activity index (SLEDAI) < 6, might have contributed to the lack of a correlation between the IFN signature in whole blood or PBMCs and disease activity in their analyses.

It has been reported that the IFN signature is associated with autoantibody profiles in SLE patients [12, 18, 21]. Rai et al. performed RNA-seq analysis to show that multiple cytokine signaling pathways were dysregulated in anti-dsDNA-positive SLE, whereas IFN signaling was predominantly dysregulated in SLE patients positive for anti-extractable nuclear antigen (anti-ENA) antibodies [21]. The abovementioned study by Sherbiny et al. also reported a positive correlation between IFN score A and the number of positive anti-ENA antibodies [18].

Most recently, scRNA-seq analysis performed in PBMCs from 33 children with SLE demonstrated that high expression of ISGs was limited to a small number of cells within each major cell type, and expansion of these cells was evident, especially in active SLE [22]. They identified a subset of monocytes coexpressing ISGs and the proinflammatory cytokine IL1β, and this cell subset was expanded in SLE. Among conventional dendritic cells, those expressing AXL and SIGLEC6 were the main subset expressing ISGs. These two subsets remained during mycophenolate mofetil (MMF) treatment, which might reflect resistance to current conventional therapies. Among B cells, a subset of extrafollicular, double-negative (DN2) memory B cells [23] showed expansion in SLE and high expression of ISGs. This study revealed molecular heterogeneity in SLE patients at the single-cell level.

## Key gene signatures other than IFN signatures in SLE

Recent clinical trials of biologics targeting type I IFN signal showed efficacy in SLE [24–26]. In a phase IIb trial of anifrolumab, a human monoclonal antibody to type I IFN receptor subunit 1, patients with high IFN signatures at baseline showed more favorable responses [25]. However, the following phase III studies revealed that high IFN signatures did not always explain more favorable response to type I IFN inhibitors [26, 27]. Clinical trials of other biologics such as rituximab, an anti-CD20 monoclonal antibody, and abatacept, a cytotoxic T lymphocyte-associated antigen 4 (CTLA4)–immunoglobulin fusion protein, failed to achieve the primary endpoints in SLE or LN patients [28–31]. During the past 60 years, belimumab, a monoclonal antibody against B cell-activating factor (BAFF), has been the only approved treatment developed for SLE [32]. These results elucidate the need for optimization of treatment strategies for SLE patients based on precise molecular profiles.

As mentioned above, transcriptomic analysis revealed several key molecular pathways in SLE. Plasma cell- and neutrophil-annotated modules were overexpressed as well as IFN signature genes, whereas ribosomal proteins and T cell signatures, including T cell surface markers and molecules expressed by lymphoid lineage cells, were repressed in SLE [13]. The neutrophil modules positively correlated with disease activity, whereas the ribosomal proteins and T cell modules negatively correlated. Pascual and colleagues longitudinally profiled 924 blood transcriptomes in 158 pediatric SLE patients [33]. Among previously defined blood modules, they found that plasmablast, cell cycle, neutrophil, histone, and B cell modules were correlated with disease activity as well as 3 IFN modules. Cross-ethnic comparisons revealed that plasmablast, cell cycle, and erythropoiesis modules were especially enriched in African-Americans, while Hispanics and Caucasians showed enrichment of neutrophil, myeloid lineage, and inflammation-related modules. They also showed activation of IFN, plasmablast, and B cell signatures, even in patients with milder disease activity (only serological or mucocutaneous/musculoskeletal activity), whereas neutrophil, myeloid lineage, and inflammation signatures were only enriched in those with renal involvement, supporting the gradual disease progression model of SLE. In LN, MMF-treated proliferative LN exhibited upregulation of myeloid lineage and inflammation modules and downregulation of B cell and plasmablast modules. Conversely, MMF-treated membranous LN showed high expression of neutrophil and IFN response modules, possibly reflecting a different etiology between these two LN subtypes. Finally, personalized longitudinal immunomonitoring revealed that different signatures were better correlated with the SLEDAI among individuals, and SLE patients were stratified into 7 subgroups based on 5 immune modules which correlated with SLEDAI  (erythropoiesis, myeloid lineage/neutrophils, plasmablast, lymphoid lineage, and IFN). These findings highlight the molecular heterogeneity of SLE and the need for personalized treatments for this disease. In another study, the same group evaluated blood microarray data from 92 SLE and 43 healthy women during pregnancy [34]. In the healthy women and SLE patients with uncomplicated pregnancies, multiple immune signatures, such as IFN response and plasma cell signatures, were downregulated, whereas erythropoiesis, neutrophil, and myeloid inflammation signatures were upregulated compared with non-pregnant women. However, the plasma cell and IFN signatures were not downregulated in the SLE patients with fetal complications, and those with preeclampsia showed remarkable upregulation of a neutrophil signature during early pregnancy.

Panousis et al. performed whole-blood RNA-seq analysis in 142 SLE patients and 58 healthy volunteers [35]. They found expression of an SLE “susceptibility signature” in patients during clinical remission, whereas an SLE “activity signature,” determined from a comparison of gene expression between inactive and active SLE patients, was dominated by genes related to oxidative phosphorylation, ribosomes, and the cell cycle. In addition, altered differential mRNA splicing associated with immune genes was observed in SLE. Patients with active nephritis were characterized by neutrophil activation and humoral response signatures. By combining the RNA-seq and genotyping data, they found that approximately 17.5% of the genes differentially expressed between the SLE patients and healthy volunteers had an expression quantitative trait locus (eQTL). They identified co-localization of eQTL top variant and SLE GWAS variant in 9 genes (*UBE2L3*, *HLA-DRB5*, *RP11*-*356I2.3*, *BLK*, *FAM167A*, *NADSYN1*, *RP11-660L16.2*, *ALDH2*, and *ALDH18A1*), and 13 of the 26 genes with perturbations in both expression and splicing in SLE had a splicing QTL.

Consistent with these findings, neutrophil dysregulation has been shown to play a crucial pathogenic role in SLE. A proinflammatory subset of neutrophils, low-density granulocytes (LDGs), enhance neutrophil extracellular traps, leading to endothelial damage and vascular dysfunction [36]. In a recent scRNA-seq study of lupus PBMCs, Minstry et al. demonstrated that ISG expression was highest in LDGs among the cell subsets evaluated [37]. In addition, intermediate-mature CD10^+^ LDGs exhibited the strongest proinflammatory phenotype and the most significant association with organ damage compared with the other cell subsets.

Petri et al. evaluated BAFF, plasma cell, and LDG gene expression signatures, as well as IFN signatures, in 243 SLE patients [16]. They found that these signatures were relatively stable in patients over time, and changes in BAFF expression did not coincide with changes in disease activity. On the other hand, the plasma cell signature was correlated with serological activity, such as increased anti-dsDNA antibody titers and decreased complement levels, and the LDG signature was correlated with elevated serum complement levels.

As most of these transcriptome data were acquired from whole blood or bulk PBMC samples, the results may have been influenced by differences in the relative abundances of each leucocyte subpopulation. Lyons et al. reported that the transcriptome data obtained from purified leucocytes, such as CD4^+^ T cells and monocytes, revealed cell-type specific gene expression profiles in SLE, which improved discrimination among SLE patients, vasculitis patients, and healthy controls, compared with transcriptome data obtained from bulk PBMCs [38]. McKinney et al. found increased CD4^+^ T cell costimulation and reduced CD8^+^ T cell exhaustion signatures in autoimmune diseases such as antineutrophil cytoplasmic antibody-associated vasculitis and SLE [39]. They identified that an exhaustion signature in CD8^+^ T cells, which was associated with poor outcomes in patients with viral infections, was correlated with a lower risk of disease relapse in cytoplasmic antibody-associated vasculitis and SLE patients.

The abovementioned recent scRNA-seq analysis in PBMCs from 33 children with SLE identified expression profiles of monogenic lupus-related genes at the single cell level [22]. Monogenic lupus-related genes were highly expressed in antigen-presenting cells, which showed expansion, especially in active SLE.

These kinds of cell-type specific transcriptome or scRNA-seq data might provide insights into distinct cell subpopulations as potential therapeutic targets for SLE.

## Disease-relevant signatures and cell subpopulations in the skin, synovium, and kidney of SLE patients

Considering that SLE involves multiple organs, transcriptome analyses must be performed in individual tissues, such as skin, synovium, and kidney, to understand SLE expression signatures. Chong et al. observed CD163^+^ macrophage polarization in discoid lupus erythematosus [40] and increased expression of M1 macrophage-associated genes, including *CXCL10* and *CCL5*, in the skin of discoid lupus erythematosus patients. In the synovium, Toukap et al. investigated gene expression profiles in synovial tissue biopsied from the swollen knee joint in SLE patients [41]. Compared with osteoarthritis and RA, SLE arthritis was characterized by increased expression of IFN-inducible genes and decreased expression of genes related to extracellular matrix homeostasis.

LN is a common manifestation of SLE and a major contributor to morbidity and mortality. An early microarray study conducted in the glomeruli of patients with proliferative LN revealed considerable heterogeneity in gene expression among samples, resulting in identification of 4 main clusters representing B cell, myeloid lineage, fibroblast/epithelial cell proliferation, and type I IFN-inducible gene signatures [42]. While fibrosis-related signature, the genes related to extracellular matrix organization pathways, was correlated with glomerulosclerosis, the type I IFN-inducible signature was associated with milder pathological features. Recently, important scRNA-seq studies conducted in the kidneys and skin of LN patients were supported by the Accelerating Medicines Partnership (AMP) in SLE Network, a public–private partnership created to develop new ways of identifying and validating promising biological targets for diagnostics and drug development. In 2017, Der et al. applied scRNA-seq to renal and biopsy tissues from 16 LN patients [43] and found that, in renal tubular cells, the IFN score was positively correlated with urinary protein levels, a pathological chronicity index and glomerular immunoglobulin G deposition. Patients with a complete response to treatment at 12 months after biopsy had significantly lower IFN scores compared with patients without a complete response. ISGs were upregulated in keratinocytes dissociated from non-lesional, non-sun-exposed skin in LN patients compared with healthy controls. In their subsequent study conducted for 21 kidney and 17 skin samples, correlations between tubular cell and keratinocyte IFN scores and between tubular cell and keratinocyte fibrosis scores were identified [44]. They also demonstrated that the fibrotic gene signature (namely *COL1A2*, *COL1A1*, *COL14A1*, and *COL5A2*) related to extracellular matrix organization pathways is a potential marker of a poor treatment response. Gene expression comparisons among proliferative, membranous, and mixed LN patients suggested activation of distinct inflammation and fibrosis pathways in tubular cells among the LN subtypes. For instance, increased type I IFN and tumor necrosis factor signaling was detected in tubular cells from proliferative compared with membranous LN patients. In another study, the same authors evaluated 21 leucocyte subsets in the kidneys of 21 LN patients, including several subpopulations of myeloid, T, NK, and B cells [45]. Using trajectory analysis, they revealed that inflammatory CD16^+^ monocytes gradually acquired phagocytic function and ultimately differentiated into M2-like macrophages in the kidney, which express high levels of various chemokine ligands and thus might coordinate immune cell trafficking in LN pathogenesis. They also found downregulated expression of exhaustion markers in kidney-infiltrating cytotoxic CD8^+^ T cell subpopulations and identified a DN2-cell-like population of B cells in the kidney. In 2020, the same group performed an integrated analysis of their renal scRNA-seq data with urinary proteomic data using 1000 urinary protein biomarkers in 30 LN patients [46]. They found that a chemokine gradient induced by IFNγ distinguished proliferative LN from membranous LN, and that urinary chemokines were produced predominantly by infiltrating CD8^+^ T cells, followed by NK and myeloid cells. These studies provided a number of insights into LN pathogenesis and patient stratification based on LN histological class and treatment response.

## Conclusions

Here, we reviewed transcriptomic studies conducted in the blood and tissues of SLE patients (Table 1). Whole-blood microarray and RNA-seq studies have revealed the contribution of IFN family members other than IFNα, including IFNβ and IFNγ, to the pathogenesis of SLE. In addition to IFN signatures, myeloid lineage/neutrophil and plasma cell signatures were shown to be correlated with distinct clinical phenotypes, such as disease activity and LN, although some of these findings remain controversial. Discrepancies in the findings among studies might be due to differences in analytical methods, treatment status, and, most importantly, the relative abundances of each leucocyte subpopulation. Transcriptome analysis of different purified cell subsets revealed cell-type-specific gene expression profiles. Further studies involving scRNA-seq analysis and combined evaluations of blood and tissue samples would be beneficial to elucidate the molecular heterogeneity of SLE and identify SLE-relevant cell subpopulations, which could provide novel treatment targets for SLE.

**Table 1:** Summary of recent transcriptome studies in SLE

Groups	Authors	Year	SLE samples and patients	Material	Method	Summary of findings
Pascual and colleagues	Chiche et al. [17]	2014	157 samples/ 62 adults	Whole blood	Microarray	Modular repertoire analysis identified three distinct IFN signatures in SLE, which involved the previous IFNα signature as well as IFNβ and IFNγ signatures.
	Banchereau et al. [33]	2016	924 samples/ 158 children	Whole blood	Microarray	Longitudinal immunomonitoring stratified SLE patients into seven groups based on five modules correlated with SLEDAI (erythropoiesis, IFN, myeloid lineage/neutrophils, plasmablasts and lymphoid lineage modules).
	Hong et al. [34]	2019	92 pregnant women	Whole blood	Microarray	Sustained IFN response and plasma cell signatures were observed in pregnant SLE patients with fetal complications. Patients with preeclampsia showed remarkable upregulation of the neutrophil signature.
	Nehar-Belaid et al. [22]	2020	33 children and 8 adults	PBMCs	scRNA-seq	The high IFN signature was limited to a small number of cells within each major cell type. Subpopulations enriched in ISGs and/or monogenic lupus-associated genes were expanded, especially in active SLE.
Other groups	McKinney et al. [39]	2015	23 adults	CD4+ T cells, CD8+ T cells	Microarray	The exhaustion signature in CD8^+^ T cells, which was associated with poor outcomes in patients with viral infections, was correlated with a lower risk of disease relapse in AAV and SLE patients.
	Chong et al. [40]	2015	9 adults with DLE	Skin	Microarray	CD163^+^ macrophages were polarized in DLE skin. M1 macrophage-associated genes such as *CXCL10* and *CCL5* were upregulated in DLE skin.
	Sherbiny et al. [18]	2018	114 adults	PBMCs and sorted monocytes, T, NK and three B cell subsets	qRT-PCR	IFN score A was increased in SLE only, whereas IFN score B was increased in both SLE and RA. Both scores were correlated with mucocutaneous and hematological, but not musculoskeletal, activity in SLE.
	Petri et al. [16]	2019	243 adults	Whole blood	Microarray	IFN, BAFF, plasma cell and LDG signatures were relatively stable in patients over time. The changes in IFN and BAFF signatures did not coincide with changes in disease activity.
	Panousis et al. [35]	2019	142 samples/ 142 adults	Whole blood	RNA-seq	The SLE “activity signature” included genes related to immune cell metabolism, protein synthesis and proliferation. Active nephritis was characterized by neutrophil and humoral response signatures.
	Catalina et al. [20]	2019	More than 1000 patients from multiple publicly available datasets	PBMCs, whole blood, skin, synovium, kidney, monocytes, B cells and CD4+ T cells	Microarray	The IFN signature was enriched in SLE skin and synovium and, to a lesser extent, in LN kidneys. The lack of correlation between the IFN signature in PBMCs and SLEDAI is due to its persistent overexpression in inactive SLE monocytes.
	Minstry et al. [37]	2019	11 adults	PBMCs	RNA-seq, scRNA-seq	The highest ISG expression was observed in LDGs among various cell subpopulations. CD10^+^ LDGs showed the strongest proinflammatory phenotype and the most significant association with organ damage.
Accelerating Medicine Partnership in SLE Network	Der et al. [43]	2017	16 adults (kidney) and 12 adults (skin) with LN	Kidney and skin	scRNA-seq	IFN scores in renal tubular cells were positively correlated with the chronicity index, urinary protein levels and glomerular IgG deposition. Patients with higher IFN scores were less likely to respond to treatment.
	Der et al. [44]	2019	21 adults (kidney) and 17 adults (skin) with LN	Kidney and skin	scRNA-seq	The fibrotic gene signatures related to ECM pathways in tubular cells predicted a poor treatment response. Comparisons among histological subclasses revealed distinct inflammation and fibrosis pathways in each subclass.
	Arazi et al. [45]	2019	21 adults with LN	Kidney	scRNA-seq	CD16+ monocytes acquired phagocytic function and differentiated into M2-like macrophages in the kidney of LN patients. Twenty-one cell subpopulations including cytotoxic CD8^+^ T cells and DN2-like B cells infiltrated the kidney.
	Fava et al. [46]	2020	30 adults with LN	Kidney and urine	scRNA-seq + proteomics	A chemokine gradient induced by IFNγ distinguished proliferative LN from membranous LN. Urinary chemokines were predominantly produced by infiltrating CD8+ T cells, followed by NK and myeloid cells.

Among the studies cited in the manuscript, the studies published since 2014 are listed

*AAV* anti-neutrophil cytoplasmic antibodies-associated vasculitis, *BAFF* B cell activating factor, *DLE* discoid lupus erythematosus, *DN2* double negative memory B cells 2, *ECM* extracellular matrix, *IFN* interferon, *IgG* immunoglobulin G, *LDGs* low-density granulocytes, *LN* lupus nephritis, *NK* natural killer, *PBMCs* peripheral blood mononuclear cells, *qRT*-*PCR* quantitative reverse-transcription polymerase chain reaction, *RA* rheumatoid arthritis, *RNA*-*seq* RNA-sequencing, *scRNA*-*seq* single-cell RNA-sequencing, *SLE* systemic lupus erythematosus, *SLEDAI* SLE disease activity index

## Data Availability

Not applicable.

## Abbreviations

AMP: Accelerating medicines partnership
Anti-dsDNA: Anti-double-stranded DNA
BAFF: B cell activating factor
CTLA-4: Cytotoxic T lymphocyte-associated antigen 4
DN2: Double negative memory B cells 2
eQTL: Expression quantitative trait locus
GWAS: Genome-wide association study
IFN: Interferon
ISG: Interferon-stimulated genes
LDGs: Low-density granulocytes
LN: Lupus nephritis
MMF: Mycophenolate mofetil
NK: Natural killer
PBMCs: Peripheral blood mononuclear cells
RA: Rheumatoid arthritis
RNA-Seq: RNA-sequencing
scRNA-Seq: Single-cell RNA-Sequencing
SLE: Systemic lupus erythematosus
SLEDAI: The systemic lupus erythematosus disease activity index
